# An automatic tracking method to measure the mandibula movement during real time MRI

**DOI:** 10.1038/s41598-024-74285-9

**Published:** 2024-10-15

**Authors:** Jérémy Mouchoux, Florian Sojka, Philipp Kauffmann, Peter Dechent, Philipp Meyer-Marcotty, Anja Quast

**Affiliations:** 1 Poliklinik für Kieferorthopädie, University Medical Center Goettingen (UMG), Robert Koch-Str. 40, 37075 Göttingen, Germany; 2 Clinic for Oral and Maxillofacial Surgery, University Medical Center Goettingen (UMG), Robert Koch-Str. 40, 37075 Göttingen, Germany; 3 MR-Research in Neuroscience, Institut für Kognitive Neurologie, University Medical Center Goettingen (UMG), Robert Koch-Str. 40, 37075 Göttingen, Germany

**Keywords:** rtMRI, Temporomandibular joint, Condyle, Tracking, LMS algorithm, Oral anatomy, Image processing, Dentistry, Medical imaging

## Abstract

Mandibular movement is complex and individual due to variations in the temporomandibular joint (TMJ). Consequently, patient-centered dentistry should incorporate patients’ specific anatomy and condylar function in treatment planning. Real-time magnetic resonance imaging (rt-MRI) visualizes relevant structures and tracks mandibular movement. However, current assessments rely on qualitative observations or time-consuming manual tracking, lacking reliability. This study developed an automatic tracking algorithm for mandibular movement in rt-MRI using least mean square registration (LMS) and compared it to manual tracking (MT) during mouth opening. Ten participants with skeletal class I underwent rt-MRI (10 frames/s). The same operator tracked the condylar pathway for the two methods, setting 2000 landmarks (2 landmarks x100 frames x10 participants) for MT and 210 landmarks (3 landmarks x7 frames x10 participants) for LMS. Time required, superimposition error, and the distance between tracked condylar pathways were compared between methods. LMS tracking was 76% faster and showed significantly better superimposition (0.0289 ± 0.0058) than MT (0.059 ± 0.0145) (*p* = 0.002). During one-third of the movement, the pathways tracked by both methods were more than 1 mm and 1° apart. These findings highlight the benefits of automatic condylar movement tracking in rt-MRI, laying the groundwork for more objective and quantitative observation of TMJ function.

**Supplementary Information:**

The online version contains supplementary material available at 10.1038/s41598-024-74285-9.

## Introduction

Digitalization in dentistry is on the rise, and the use of three-dimensional (3D) static imaging is increasing steadily. This offers the opportunity to assess the anatomy of each patient individually and enables dentists to build patient-specific 3D models, which can be used to plan orthognathic surgery, prosthodontic or endodontic treatment^1–3^. Most of these models provide high accuracy and precision in the area of the teeth and the bones but lack information regarding the temporomandibular joint (TMJ) with its muscles, ligaments, and articulation. This bears the risk of missing structural and functional characteristics of the individual TMJs and could be an obstacle to the goal of patient-centered dentistry.

The TMJ is a bilateral joint performing two distinct motions during mandibular movement: (1) Mandibular rotation around a transverse horizontal axis, and (2) translational motion in which the condyle and the disc move together beneath the articular eminence. These movements occur between 2000 and 2500 times per day and are involved in every motion of the lower jaw like chewing, swallowing, and speech^4^. In consequence, impairment or unintentional iatrogenic changes of TMJ function may have a high impact on daily life and patients’ well-being^5^. Therefore, considering the high individuality of condylar movement^6,7^ and implementing its complexity in patient-specific 3D models might improve treatment outcomes in orthognathic surgery or prosthodontics^8^. A popular method to track TMJ function and condylar movement is computerized axiography. The devices enable the recording of the condylar pathway with high precision^7^, but do not provide the patient’s anatomical structure with the individual position and shape of the moving condyle. To gain this information, axiography has to be combined with imaging techniques like cone beam computed tomography or computerized tomography^9^. However, the ALARA principle (as low as reasonably achievable) restrains the number of patients who can benefit from imaging methods.

One possibility to acquire high-quality images of the TMJ and its motion without using X-rays is real-time magnetic resonance imaging (rt-MRI)^10–12^. With the use of higher framerates^13,14^, multiple slices^15,16^, and algorithms more robust to metal-caused artifacts^17^. the technique further improved in recent years.

Unfortunately, the use of real-time MRI is limited until now to qualitative observation^18,19^ or the manual tracking of the condylar pathway relying on the placement of two landmarks at each frame of the acquisition^6^. In addition to being time-consuming for the rater, this method suffers from a lack of reliability due to the unreliable manual placement of the landmarks on the frames of the real-time MRI^20^. Yet, the robustness of the tracking is essential for the computation of the instantaneous center of rotation, which suffers from any translational error^21^.

Therefore, this pilot study aims to propose a method for automatic tracking of the condylar pathway using rt-MRI and a least mean square (LMS) registration with two goals: (1) to improve the accuracy of the tracking, and (2) to reduce the amount of time required for the operator to track the mandibular movement. To assess if these goals could be achieved, ten participants with skeletal class I underwent rt-MRI (10 frames/s) during moth opening and closing. The condylar pathway was tracked using LMS and manual tracking while the time required, the superimposition error, and the distance between the tracked condylar pathways was analyzed.

## Results

All 10 participants could perform the mouth opening-closing cycle within 10 s and both, the manual and the automatic methods, could track the condylar movement sufficiently. The operator placed 2000 (2 landmarks x10sec x10 frames per sec x10 participants) landmarks for the manual method and 210 (3 landmarks x7 frames x10 participants) landmarks for the automatic method. Footage of the moving condyle is available in the complementary resources (supplementary Video 1 & 2).

### Results I—Reduction of the duration of the pre-processing operations

**Fig. 1:**
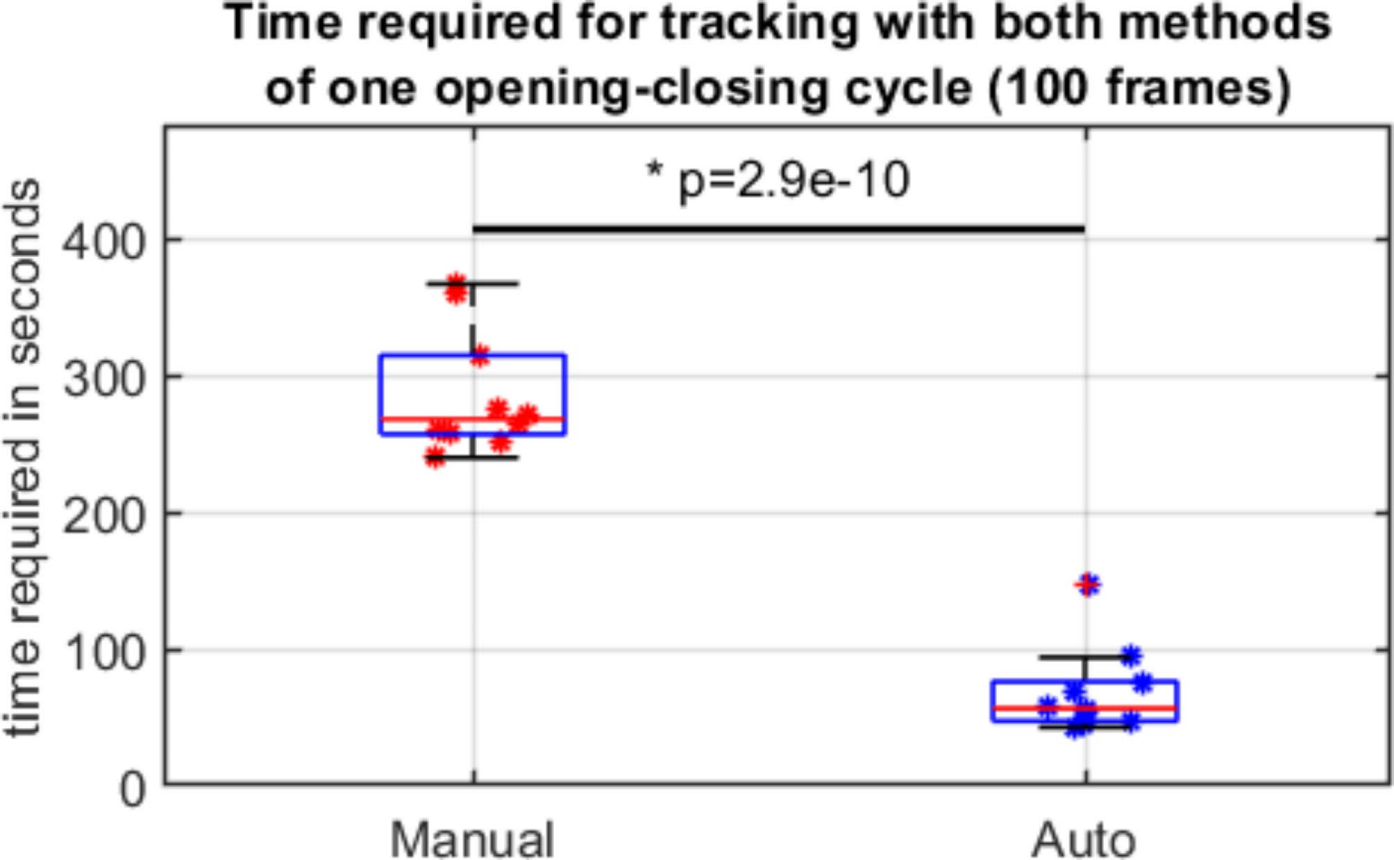
Comparison of the time required for the operator to track the mandible using both methods.

The operations required by the two tracking methods were different. The average duration required for manual tracking (landmark placement) was 267.0 ± 57.7 s and 56.4 ± 28.9 s for the automatic method (including the drawing of the area of interest and the landmark placement), meaning a significant improvement of 76% (*p* < 0.005) (See Fig. 1).

### Results II—Reduction of the error of superimposition

**Fig. 2:**
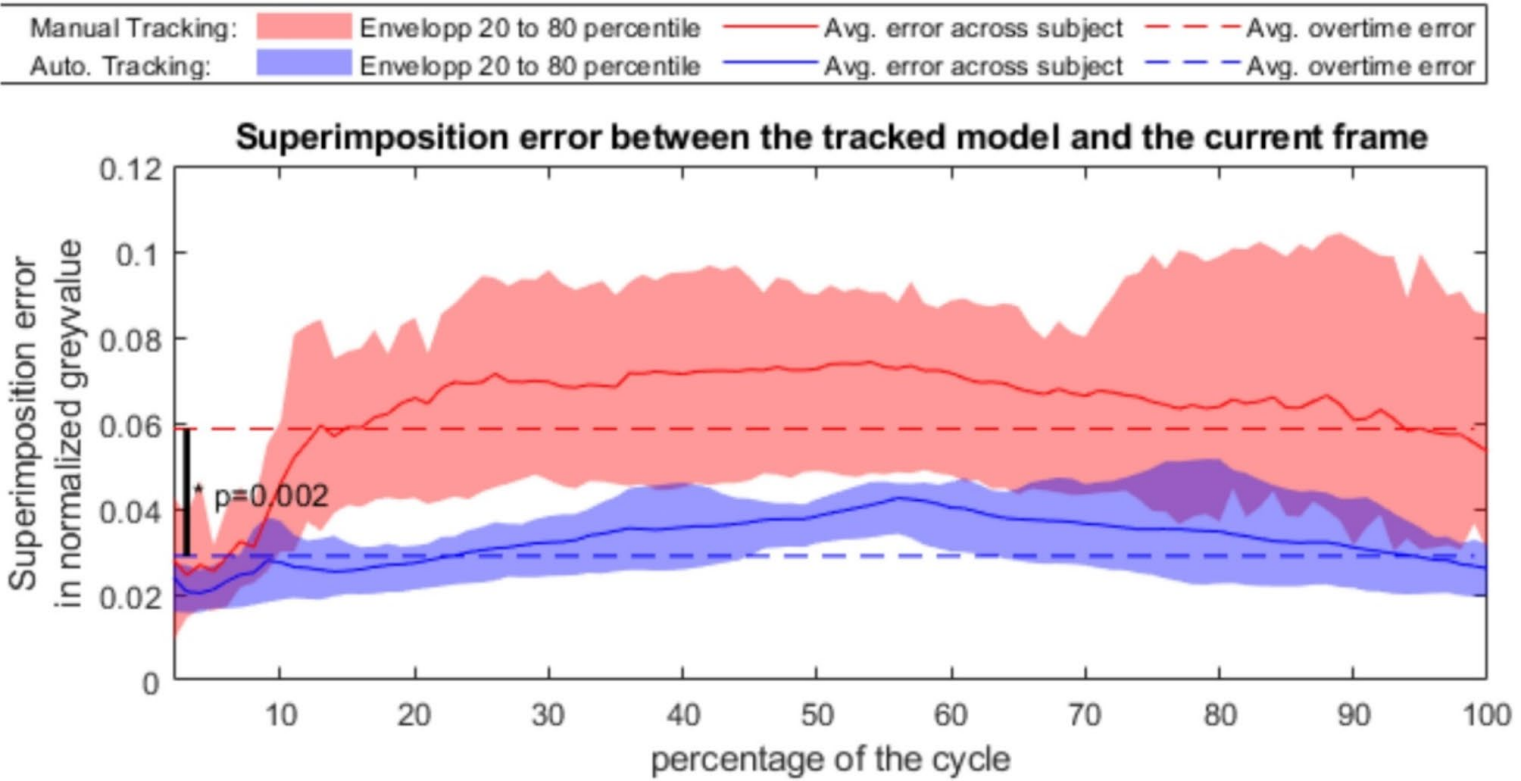
Comparison of the error of superimposition obtained with both methods. The automatic tracking is represented in blue while the manual tracking is in red. Its evolution frame per frame averaged across every scan is represented as a plain line and an envelop of lighter color represent the 20 to 80 percentile of the population. Eventually, the averaged error of superimposition across frame and subject is represented as a broken line.

The automatic tracking method shows a significant reduction of the error of superimposition, from 0.064 ± 0.019 with the manual method to 0.028 ± 0.008 (*p* < 0.005), as illustrated in Fig. 2. This error of superimposition is the lowest at the start of the movement cycle. When manually tracking, the overlap error rapidly increases to reach a plateau. For the automatic tracking, the error increases slowly over time to reach its maximum in the region of the MMO position and then slowly decreases.

### Results III—Significantly different landmarks’ pathways

**Fig. 3:**
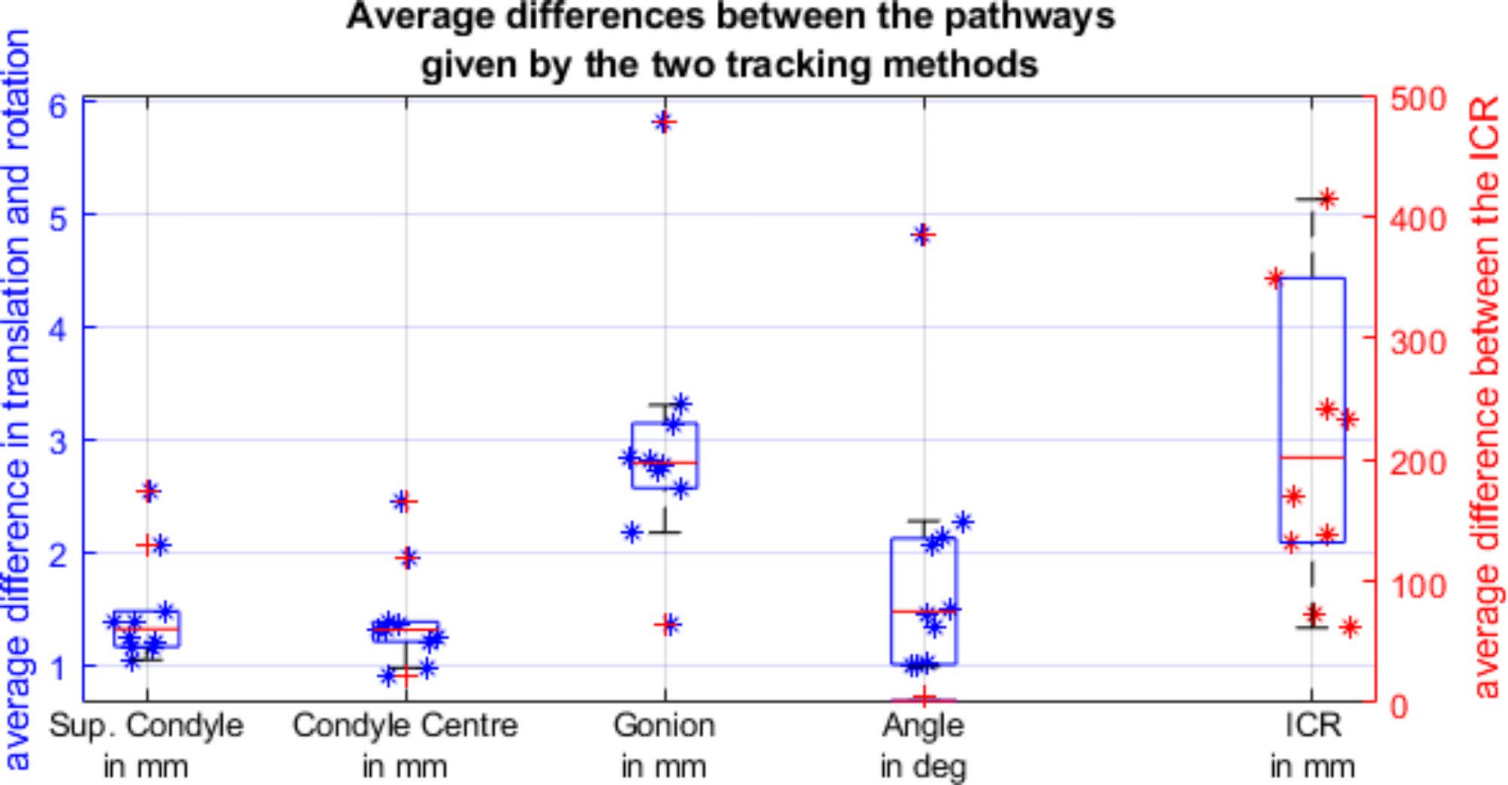
Differences between the results of the two tracking methods when assessing the path of the superior condyle, the center of the condyle, the angle to the original inclination, and the path of the ICR.

When comparing the pathways given for both methods (illustrated in Fig. 3), we found that the four landmarks, the condyle superior, condyle’s center, the gonion, and the instantaneous center of rotation (ICR), were significantly further apart than 1 mm during at least one third of the movement (*p* < 0.05) with upper third medians of 1.31 ± 0.32 mm, 1.31 ± 0.18 mm, 2.79 ± 0.58 mm and 169 ± 151mmrespectively. The difference in inclination between the two methods was significantly greater than 1° with an upper-third median of 1.47 ± 1.12°.

## Discussion

**Fig. 4:**
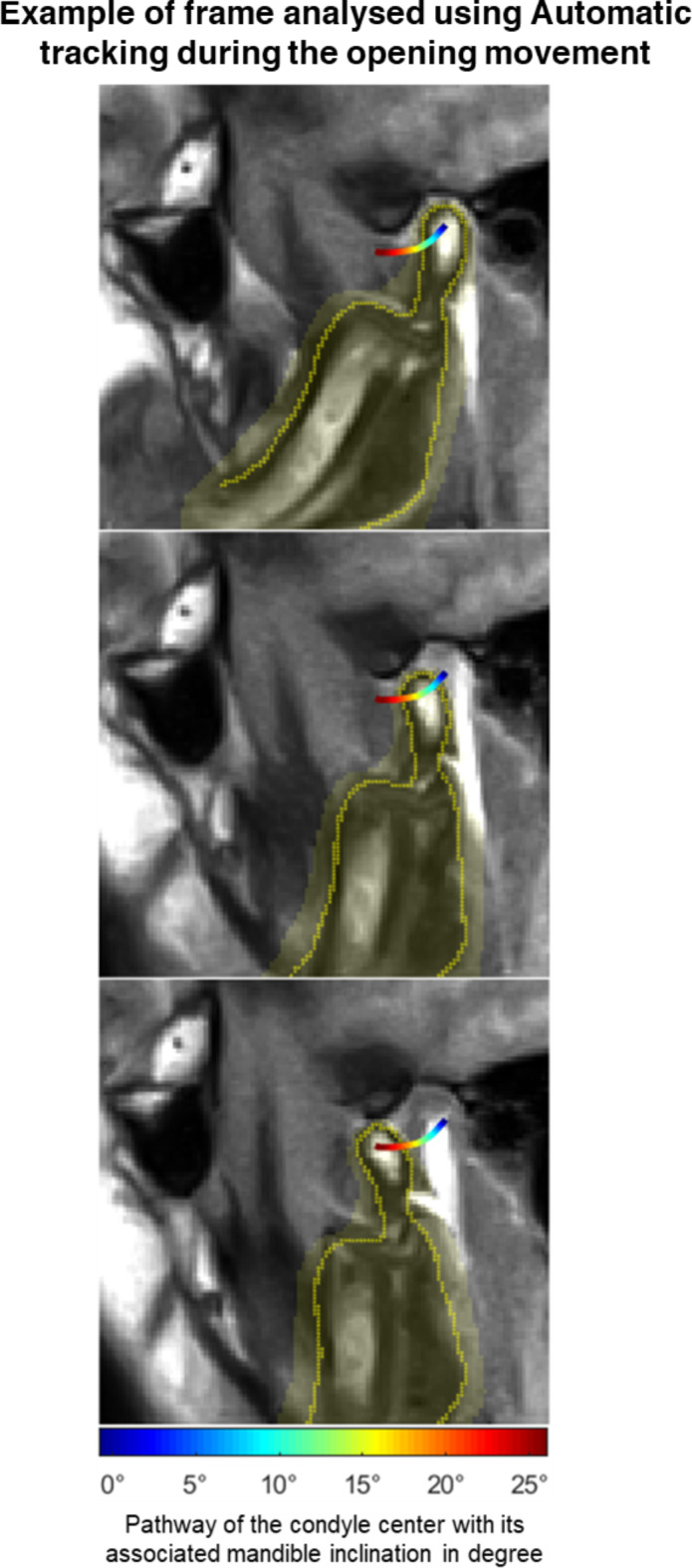
Movement of the Jaw according to the automatic tracking. Three frames (0%, 70% 100%) of the opening movement are displayed. The area of the jaw is highlighted in yellow. The contour of the jaw predicted by the tracking is displayed as yellow dots. The path of the center of the condyle is colored according to the inclination of the jaw.

To the best of our knowledge, the LMS registration presented in this study is the first automatic tracking algorithm for the condylar pathway acquired by rt MRI. It follows the movements of the TMJ frame by frame and brings valuable information to the practician as illustrated in Fig. 4. In contrast to the MT method, this algorithm does not only rely on the landmarks manually placed by the user on one of the acquired slices but uses all the pixels of the area drawn by the user in several slices. This leads to much more information for the algorithm to track the mandibular movement.

Because the LMS method does not use manual landmarks for the final transformation and can interpolate the position of the landmarks between the keyframes, it significantly reduces the number of landmarks to be placed, which is a long and tedious process. Furthermore, it requires less precise landmark placement and, as a consequence, less concentration of the user. This potential for time-saving not only increases the value of condylar rtMRI for clinical application but also enables future studies to increase the number of included participants and the number of movement’s cycles analyzed.

As demonstrated, the error of superimposition was significantly reduced by LMS compared to MT. The error of superimposition is based on the distance between the pixels of the model and those of the data at the current frame, projected on the tangent of the pixel. This has the consequence that pixels having vertical tangent because they are in a surrounding where the grey value does not vary a lot (center of the bone, for example) will have less impact than the pixels having an oblique tangent due to a surrounding with high contrast (side of the bone for example). Thus, the lower superimposition error obtained by the automatic tracking is the consequence of better tracking of the contours of the transformed mandibula. This result was to be expected since the algorithm was specifically designed to minimize this error.

Obviously, the superimposition error does not replace the comparison of automatic tracking with external motion tracking. This metric takes the first frame of the acquisition as a model and supposes that the bone observed in the first frame does not change in shape during the movement. Yet, when moving, the section of the jaw observed on the slices slightly changes its shape, (1) due to the blurriness of the movement and (2) due to the inclination of the acquisition plane, not perfectly perpendicular to the axis of the movement. Since this deformation is not linear, it cannot be completely compensated by the scaling applied during the automatic tracking. The superimposition error is then the distance between two slightly different shapes, resulting in an unavoidable error and introducing a small bias in the metric.

This bias can be observed by the increasing superimposition error during the movement, reaching its maximum at the maximum mouth opening (MMO) position and then reducing during the mouth closing while the observed jaw section regains its shape. The low variance of the error of superimposition over the whole movement indicates that this behavior is relatively constant across the analyzed scans, meaning that the change of shape of the jaw over time is similar for every participant.

On the other hand, manual tracking produces a superimposition error, which quickly increases at the start of the movement and remains relatively constant during the cycle. This difference in behavior compared to the automatic tracking can be explained by the bigger misplacement of the tracked jaw over the observed data using this method, which has a bigger impact on the superimposition error than the effect of the small shape’s overtime changes. The higher variability of the overlap error of the manual tracking might be interpreted as a lack of reliability of the method, probably due to the lack of reliability of the landmarks placement^20^.

The two methods differ thus in terms of the time required to use them and their superimposition error, but they also output pathways that are more than 1 mm and 1° apart. This difference is slightly above the acquisition’s resolution. The manual method’s lack of reliability and lower superimposition show that it can not be considered ground truth to evaluate accuracy. This will have to be done using external motion tracking, as discussed above and as applied by Xu et al.^22^. Once the accuracy is measured, the method presented here will be a non-carcinogenic equivalent to the combination of computerized axiography and CBCT scan, which has an accuracy slightly below 1 mm^22^.

The instantaneous center of rotation especially suffers from a considerable difference of path. This is in accordance with the results of Safrany et al.^21^ which showed that small differences in tracking lead to a big impact on the ICR due to its derivative nature. Since the path of a point and the evolution of the inclination are enough information to describe the movement of a rigid object fully, future research should use the path of the center of the condyle and the inclination of the jaw to describe the movement of the mandible. The center of the condyle, computed through the contours of the condyle, should indeed be more reliable than other landmarks manually placed on the mandibula.

This study set the foundation for a qualitative observation of the functioning of the TMJ. Some limitations remain to be solved. The method is, for now, limited to one TMJ, and the asymmetry of the movement cannot be measured. Still, by merging the different slices of the real-time acquisition and comparing them to a more detailed static MRI scan, it will be possible to improve the registration and track both TMJ simultaneously. This would also solve the deformation problem of the section of the mandibula observed in the slices and enable the observation of the asymmetry and synchroneity of the movement. Another limitation of the method is the dynamic MRI scan’s sharpness and resolution. While the settings used for the presented method are the current standards^6,23,24^, a better sharpness would also benefit both manual and automatic methods. A more powerful MRI would reduce the slice thickness while keeping enough contrast. Another limitation of this study is the low sample size. The positive preliminary results presented above enable us to compute the effect size for future studies and support the use of a bigger sample size in the future. Eventually, since the manual placement of the landmarks does not need to be exact, algorithms using artificial intelligence such as the one developed by Eslami et al.^25^ could be used to place the landmarks and completely automatize the process roughly. The observation of the movement of the mandible in relation to the rest of the skull will provide more information and support to the dynamic jaw models already present in the literature^26^ for a deeper understanding of the complex interaction of each ligament, each muscle, and the physical constraints applied to every structure.

## Method

### Participants and real-time MRI

This study was approved by the Institutional Ethics Committee (no. 6/7/21) following the Declaration of Helsinki. Written informed consent was obtained from all participants.

Subjects were ten adult orthodontic patients (mean age: 31.5 ± 5.9 years, female: *n* = 5) with full dentition, skeletal class I (mean Wits appraisal: 0.17(± 2.06) mm), and no symptoms of temporomandibular disorder (TMD). Exclusion criteria were age below 18, craniofacial anomaly, large tattoos, and intraoral or intracorporal metal components such as orthodontic braces, pacemakers, cochlear or joint implants due to the MRI constraints.

In each participant the right TMJ was scanned using a Siemens Magneton Prisma fit with T2/T1 contrast (refocused FLASH) at ten frames per second according to a previously published protocol^20^. The in-plane resolution was 0.75 × 0.75 mm for a field of view of 128 × 128 mm. The slice thickness was set at 6.0 mm, echo time (TE) at 1.56 ms, repetition time (TR) at 2.56 ms, and the number of excitations (NEX) at 1. Three slices (6 mm of inter-slices) were positioned on the center of the condyle, localized through a static calibration scan in sagittal, coronal, and transversal planes. The acquisition plane was rotated to include the mandibular ramus. The patients were instructed to do four cycles of mouth opening and closing (intercuspal position – maximal mouth opening (MMO) – intercuspal position), each cycle lasting ten seconds. Standardized instructions on the monitor for opening and closing the mouth were displayed to the patient. Due to the time required by the manual tracking, only the first cycle of each patient was analyzed in this study.

### Pre-processing for automatic and manual tracking of the mandibular movement

The pre-processing of all data has been done by a the same dentist (F.S.) on a custom Matlab R2019a applicationrunning on an ASUS ROG G751JT with 16Go RAM and an Intel i7 processor. Through this application, a sequence of tasks was applied.

**Fig. 5:**
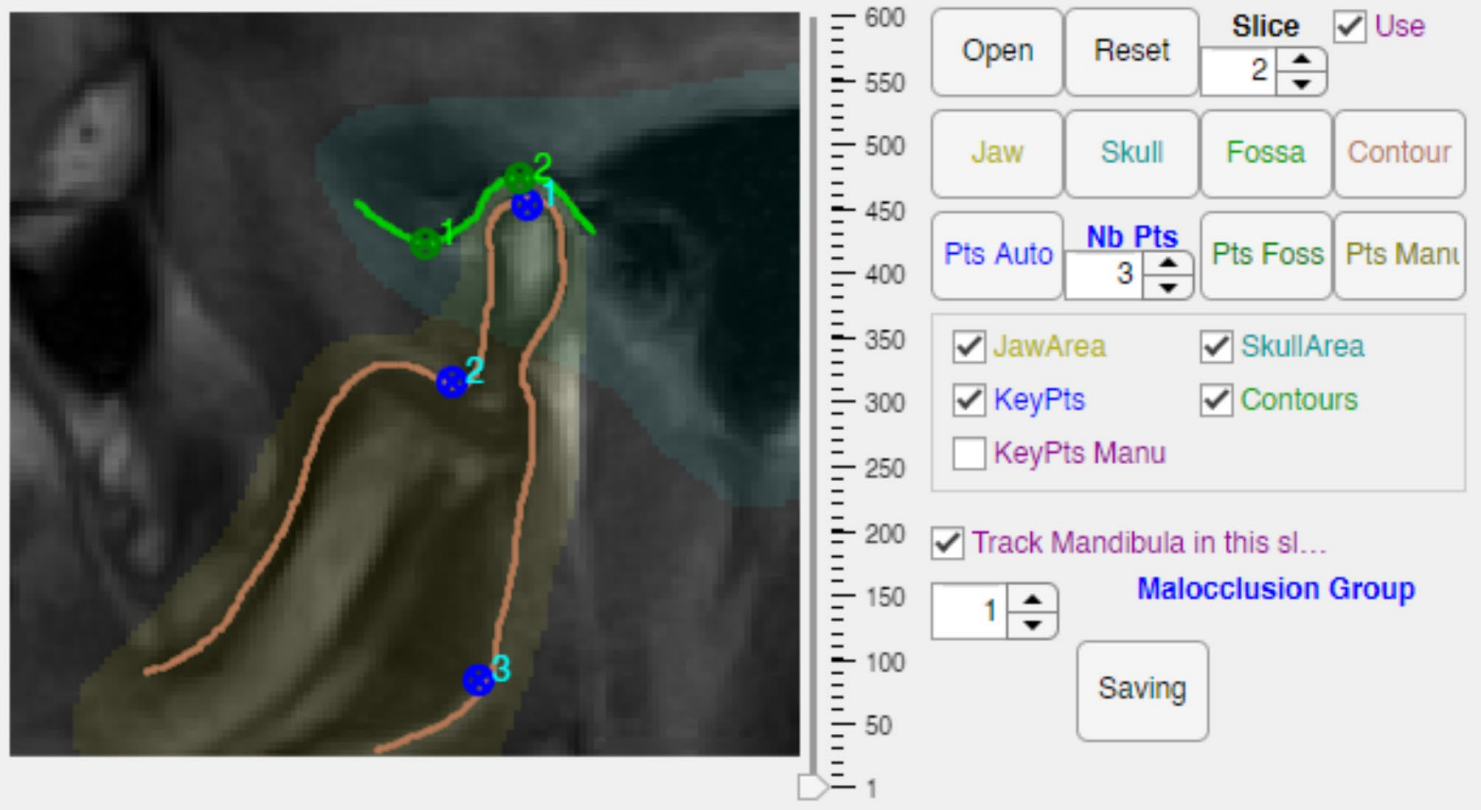
Screenshot of the GUI once the rater is finished. Manual landmarks are not displayed for visibility. They share positions 1 and 3 with automatic tracking. The areas are drawn by the rater and represent the pixels used for the registration. The contours are also drawn by the user but are only used to determine the center of the condyle. Due to the inclination of the acquisition plane, the Processus coronoideus mandibulae is not visible.

First, the contours of the condyle and the fossa articularis were marked by the same experienced user, as illustrated in Fig. 5. To ensure the precision of the position of the condyle’s center, it was not placed by the rater but defined automatically as the center of the circle fitting the contour of the condyle’s head. This circle was computed using LMS algorithm(Pratt, 1987).

Then, from the initial 100 frames, seven keyframes were selected from each MRI scan as follows:

- first and last frame of each scan.
- first and last frame of each movement cycle.
- every 25th frame in between.

On these keyframes, the operator placed two landmarks on the temporal bone (1. the most inferior point of the eminence crest and 2. the most superior point of the fossa articularis) and three landmarks on the mandible (1. the most superior point of the condyle, 2. The most inferior point of the incisura mandibulae and 3. the gonion).

### Method I—Automatic tracking of the movement

For an automatic tracking of the mandible movement in the rtMRI data a three steps procedure has to be performed based on a mathematically algorithm: (1) the rough transformation based on landmarks, (2) the pixel extraction, and (3) the LMS registration.

#### Rough transformation based on landmarks

First, the rough transformation was extracted from the manually placed landmarks at each of the frames using SVD algorithm. At each frame, the position of the landmarks was interpolated from the two closest keyframes. The draft transformation described the transformation of the landmarks from their position in the first frame to their position in the current frame using the formula:

$$\:{Y}_{rough}={T}_{rough}+{R}_{rough}*Coor{d}_{2D}$$

With Coord_2D_ the coordinates of the pixel in the scan, Y_rough_ its rough transformed position, and T_rough_ and R_rough_ the rough translation and rough rotation respectively.

#### Extraction of the coordinates and tangent of the pixel belonging to the mandibula

In order to filter the pixels of interest for the registration, the rough transformation (T_rough_, R_rough_) is applied to the mandibula at each frame to act as a mask and filter out every pixel that is not covered. The greyvalues of the masked area are then normalized to achieve two goals:

First, the grevalues are normalized to be in the same range of value as the spatial dimensions:

$$\:Scal{e}_{FirstFrame}=\frac{interdecile\left(dot\left(\overrightarrow{tangente},\overrightarrow{Coor{d}_{2D}}\right)\right)}{8*interdecile\left(G{V}_{FirstFrame}\right)}$$

With Scale_FirstFrame_ the normalization coefficient, GV_FirstFrame_ the grey values of the pixels of the mandibula area in the first frame, $\:\overrightarrow{tangente}$ the last component of the principal component analysis applied to the pixels’ coordinates, and $\:interdecile$ the difference between the first and the last deciles (from 10 to 90%).

The second goal of the normalization is to keep the grey-values of every frame in the same range. The other frames are therefore normalized using the formula:

$$\:{3}^{rd}Coor{d}_{3D}=\left(GV-D{1}_{currFrame}\right)*\frac{interdecile\left(G{V}_{FirstFrame}\right)}{interdecile\left(G{V}_{currFrame}\right)}+D{1}_{FirstFrame}$$

**Fig. 6:**
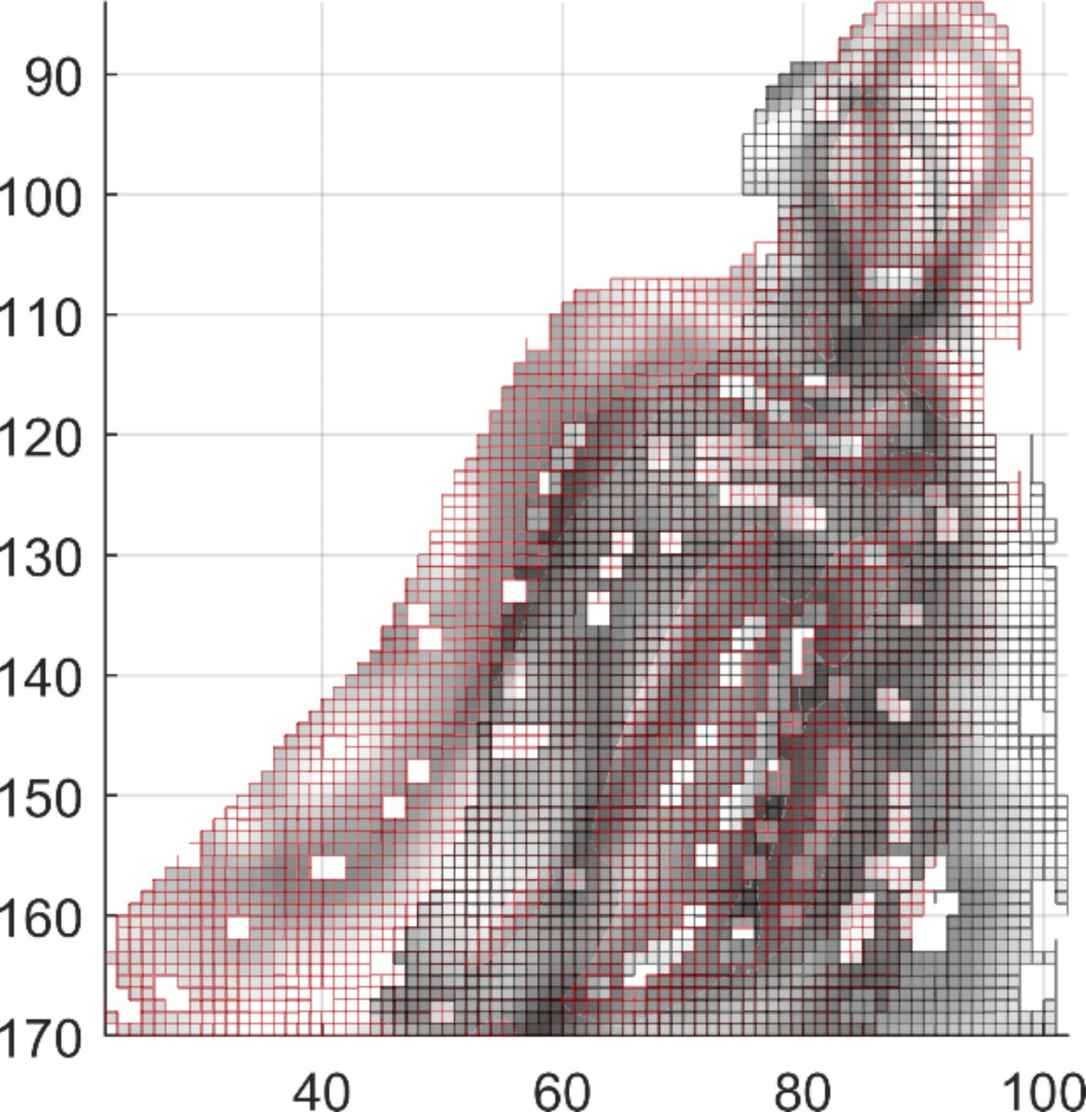
Grey-values of the pixels within the area drawn by the rater for the mandibula for the current frame in black and for the first frame in red. Horizontal pixels (with vertical tangents) are discarded to reduce computing power. [Matlab, Version R2019a, https://www.mathworks.com/].

**Fig. 7:**
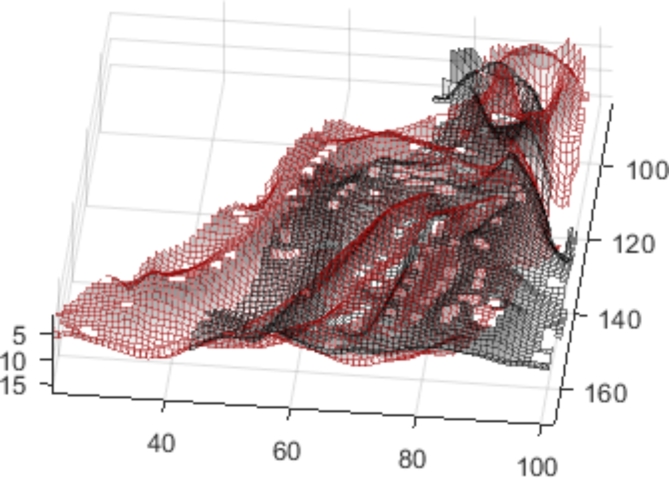
3D shape extracted from the grey-values for the current frame in black and for the first frame in red This 3D shape of the first frame will be used as a model for the automatic tracking to compute the optimal superposition. [Matlab, Version R2019a, https://www.mathworks.com/].

With 3rd Coord_3D_ the third coordinate of the 3D representation, GV the grey value of the pixel and D1_FirstFrame_ the first deciles of the grey values of the first and current frames respectively. The result is displayed by Figs. 6 and 7 Once the 3D representation of the mandibula is computed, the tangent of each of the voxels is computed as being the last component of the principal component analysis applied to the voxel and its neighbors. Finally, pixels whose tangent is too close to the vertical (< 8°) are removed to reduce the needed computational power.

#### 2D LMS-registration based on the 3D representation

Once the 3D representations of both slices are computed for every frame, the LMS registration is applied. The algorithm is designed to minimize the distance between the transformed model (mandible observed at the first frame) and the mandible observed on the current frame. This distance (the error of superimposition) is computed as the mean of the distance between each pixel of the model and its projection, following its tangent, on the observed frame. This is achieved through a repeated cycle of three parts: (1) the points pairing, (2) the rigid body transformation, and (3) the scaling. During this process, every pixel of the model does not have the same weight. In order to focus the precision of the superposition on the area of interest, the pixels of the condyle have their weight doubled, and pixels from the middle slice, centered on the TMJ, have a supplementary weight increase of 150%.

At each cycle, each pixel of the first frame is paired with the closest point from the observed scan. The average distance between each pair is called the error of superimposition Then, the rigid body transformation aims to find the translation T_LMS_ and the rotation R_LMS_ using an LMS approach to minimize the error of superimposition. Eventually, scaling aims to find the translation T_LMS_ and the homothety H_LMS,_ minimizing the mean of the squared error. Since this approach searches for a local optimum, the initial transformation is given by the rough translation previously computed. Eventually, the final 2D transformation (T_LMS_, R_RMS_, H_RMS_) can be represented as

$$\:{Y}_{LMS}={T}_{LMS}+{R}_{LMS}*{H}_{LMS}*X \quad {\text{with}}\quad {H}_{LMS}=\:\left(\begin{array}{ll}{H}_{11}&\:0\\\:0& \:{H}_{22}\end{array}\right) \;\;\; {\text{and }} \;\; {{\text{R}}_{{\text{LMS}}}} \;\;\;\; {\text{the rotation matrix}}$$

### Method II—Manual tracking

To compare our automatic tracking method to the actual state of the art, we implemented manual tracking according to Krohn et al. (2020). The same operator placed two landmarks, condylar superior and gonion, at the first frame and each frame of the movement during the first movement cycle, resulting in 200 landmarks placed on the 100 frames of the first opening - closing cycle for each participant. For each frame, the translation was computed based on condyle superior, while the rotation was adjusted using gonion.

### Analysis

For each subject and for both methods, three metrics were recorded and compared: (1) the time taken by the rater to track the mandible, (2) the error of superimposition between the two methods, and (3) the distance between the pathways of the superior condyle, the condyle’s center, the gonion and the instantaneous center of rotation (ICR) (see^6^ in the most distant third of the movement as well as the angle between the rotation of the mandibula given by both tracking methods.

The normal distribution of the data has been assessed using the Shapiro-Wilk Test. With the exception of the manual tracking method’s superimposition error, every group we compared did not follow a normal distribution (*p* < 0.05). Therefore, the Wilcoxon sign rank test has been used, with *p* < 0.05, to assess the difference between the durations and the superimposition error and if the pathways and the inclination were more distant than their thresholds (right tail). For all metrics, median and the interquartile range were reported as durations in seconds, distances in millimeters, and angles in degrees. The error of superposition is a constructed value based on distances and greyvalue and, therefore, has no dimension. The statistical assessments and the figures were computed using Matlab R2019a.

## Electronic supplementary material

Below is the link to the electronic supplementary material.

Supplementary Material 1

Supplementary Material 2

## Data Availability

The datasets generated during and/or analysed during the current study are not publicly available due to medical data protection but are available from the corresponding author on reasonable request.The matlab scripts are available only at this address: https://owncloud.gwdg.de/index.php/s/GrkqnfXYbVQ5rQM.
